# “Silent Voices”: A Description of Views and Attitudes of Health Professionals towards Reproduction by HIV Positive People

**DOI:** 10.1155/2014/205872

**Published:** 2014-08-04

**Authors:** Vezumuzi Ndlovu

**Affiliations:** Department of Development Studies, Lupane State University, 44 H. Chitepo Street, Bulawayo, Zimbabwe

## Abstract

*Objective*. The role of health professionals in the decision making process of patients is usually heard or seen from the perspective of the patients. This paper gives the usually silent and invisible health professionals voice and visibility. It describes their views and attitudes towards reproduction by couples who are HIV positive and attempts to understand their perspectives. *Methods*. In-depth interviews were conducted with twelve health professionals at an opportunistic infections clinic. Transcribed interviews were analysed using the grounded approach to identify patterns and themes concerning views and attitudes of health professionals towards reproduction by HIV positive people. *Results*. The study found that most health professionals generally had a negative attitude towards childbearing by HIV positive couples. Their views and approaches on the issue were based mainly on biomedical considerations. The main discourses on childbearing that emerged from the study were the conditional choice, the antichildbearing, and the prorights. *Conclusion*. Most of the health professionals interviewed tend to take a generally negative stance towards reproduction by people with HIV/AIDS. There is a need for a clear set of guidelines for health professionals (HPs) on how to deal with HIV positive people who may desire to reproduce.

## 1. Introduction

The AIDS epidemic which has hit Southern Africa with such devastating force affects a variety of reproductive issues among infected people. These include whether or not to become pregnant, when and which type of contraception to use, and decisions on whether to continue or try to terminate a pregnancy. Findings from studies in both developing and developed nations indicate that a significant proportion of HIV infected adults still desire or intend to have children [1–4]. With the advent of highly active antiretroviral therapy (HAART) and its impact on the health of the infected as well as its impact on lowering mother to child transmission of HIV, the number of HIV infected people considering childbearing has increased [5–7]. While much attention has been paid to the reproductive choices and intentions of the infected as well as to some factors that influence these [1–4], most studies are silent on the role of health professionals in the reproductive and sexual lives of HIV infected people.

There are few studies that mention the role of health professionals in the reproductive sphere of HIV positive people, yet these are the people who usually play a significant role in the decision making process of HIV positive people [8]. This is because of their medical expertise and that most HIV infected people make their reproductive decisions within a biomedical and health institution context [7, 9, 10]. There are some studies that have involved health professionals and they found that biomedical considerations usually dominate health care providers' attitudes towards reproduction by HIV infected people [11, 12]. Findings from this study indicate that there are three dominant discourses concerning childbearing by people with HIV among health professionals. These are the conditional choice, the antichildbearing, and the prorights discourses. The first two are rooted in the biomedical model of health where biomedical considerations are prominent while the third takes a human rights perspective.

## 2. Method

### 2.1. Study Design

Taped in-depth interviews were conducted with 12 health professionals in two opportunistic infections clinics in Bulawayo. Purposive sampling was used to recruit respondents. Health professional interviewees were selected for their involvement in the care, treatment, and counselling of HIV positive people. This research among health professionals was part of a broader study that explored reproductive decisions among HIV positive couples in Bulawayo, Zimbabwe, in 2010. Interviews with HIV positive couples revealed that people who featured most in their reproductive and sexual lives were the health professionals (HPs) (nurses, doctors, and counsellors) in opportunistic infections clinics and support groups. As a result the defining characteristic for this sample was involvement with HIV positive people in the past two years.

Using these characteristics, six counsellors, four nurses, and two doctors were interviewed by the researcher at their place of work. Informed consent was obtained from all the respondents and so was the right to tape record the interviews. All the respondents were married and had children except one male nurse. More counsellors than any other group of HPs were selected because, according to the interviews with HIV positive people, they seemed to interact with them more than the nurses or the doctors.

### 2.2. Data Analysis

Data were analysed using a grounded theory approach, based on a process that helps researchers to “discover” categories, themes, and patterns that emerge from the data. Taped interviews were transcribed in the original language and then translated into English. Transcribed interviews were then content analysed to identify patterns and themes concerning attitudes of health professionals towards reproduction by HIV positive couples. The strategies used in the data analysis were a systematic review and a thoughtful reading of interview data, coding, categorising, and sorting for patterns, and the construction of the story told.

## 3. Results

### 3.1. Information on Reproductive Issues Given to HIV Positive People in General

This study found that the majority of HPs interviewed generally gave people incomplete information on reproduction. The information given to HIV positive couples centred on pregnancy prevention and prevention of reinfection. The possibility of having children where and when it was discussed was usually presented as a “dangerous” possibility that people must try to avoid.

Responding to the question on what they tell people with HIV regarding reproduction some respondents had this to say;

*“we tell them there is re-infection when you have intercourse with an infected person so the first piece of information that we give them is the proper use of the condom…” (male counsellor).*

*“The use of the condom reduces the re-infection rate and apart from that it prevents pregnancy. If you get pregnant there are high chances of getting an HIV positive child” (nurse).*

*“what we tell them is that its not wise to be pregnant when one is HIV positive…” (nurse).*

Another counsellor also pointed out that they emphasised what he called “practical aspects” when giving HIV positive people information on their future reproductive prospects. He said they emphasised the issue of reinfection and drug resistance if people do not use condoms and the negative repercussions this has not only for them but also for the opportunistic infections clinics and others who are HIV positive.

The “practicalities” that they are made aware of are in a way meant to steer them from the path of unsafe sex and dissuade them from having children. The manner in which the said “objective information” regarding reproduction was delivered reveals the subjective prejudices of most HPs interviewed.

### 3.2. Health Professionals and HIV Positive People Who Want to Conceive

All of the counsellors and some nurses indicated that they had in the past months (before July 2010) been approached by an HIV positive individual or couple who wanted to have a child. Most HPs empathised and some sympathised with them and were aware of the social and personal challenges faced by these people. One counsellor said

*“…because you are young, you are a newly wed couple, you meet a lot of challenges and in an African situation where you are a daughter in law, you cannot be a daughter in-law without children.”*

Though aware of the sociocultural constraints faced by most of these couples or individuals, most health professionals still felt that they had to seek medical approval before they could reproduce. Some pointed out that these people could have a child provided they fulfilled certain conditions set by the health professionals (conditional choice stance). Others were of the view that though their need to have a child may be genuine it was not necessary to have a child considering their condition (antichildbearing stance). They felt the risks posed to both the mother and the child far outweighed the need to have a child.

Examining the information given to those who desire children, one can not help but notice that the information itself and the manner in which it was given were, in counselling principles, generally biased and meant to discourage childbearing. Responding to how he would deal with someone desiring to have a child, one counsellor responded this way;

*“well, our most important area of discussion is; it is still possible to be pregnant and get a child when you are HIV positive but then our area of interest is what does it mean to be pregnant when you are HIV positive…it means whilst we had built your health so much with ARVs the stress and strain related to delivery and the psychological pressure related to nursing a child may actually be counter to what the ARVs are trying to achieve….”*

Other counsellors said

*“…there is a lot that goes on when you are pregnant. Pregnancy lowers your immunity and you are then open to other infections, these other infections will also lower your CD4 cell count and increase the rate of progression to AIDS.” *

*“…you feel pity for them, really it's a young couple, they have no child, they really want to have a child but just because of this (HIV) they cannot.” *

Most HPs interviewed emphasised the negative aspects of childbearing when giving potential parents advice. Apart from the fact that the validity of this information can be disputed, the manner in which it was delivered made it seem compulsory for HIV positive people to act upon. This deviates from the professional expectation from counsellors to give clients objective, unbiased, and unprejudiced information [13].

### 3.3. Prevailing Discourses about Childbearing by HIV Positive People

The study found that there are three dominant discourses among HPs concerning reproduction by HIV positive couples or people. These are the conditional choice discourse, the antichildbearing discourse, and the prorights discourse. The study found that six of the HPs interviewed took a conditional choice stance, while four took an antichildbearing stance and only two took a prorights stance.

### 3.4. The Conditional Choice Stance

The conditional choice stance argues that HIV positive people may have children as long as they made their decision on the basis of information and advice given to them by HPs. There are a number of conditions that these health professionals indicate as necessary to be fulfilled before HIV positive people can have children. It is important to note that these conditions are to be determined by the HPs though on the other hand they claim to be neutral facilitators. The following are some of the conditions pointed out:

*“they have to do that (decision making) on an informed basis….”*

*“I think people need information, the correct information and be allowed to make choices based on correct information….”*

*“people can have children as long as they are able to make sure that they make every effort that child does not become infected….”*

*“if they want to make a decision to get pregnant they have to consult a doctor who will advise them on how big the risk of getting pregnant is.”*

*“they have to consult a medical person who will look at their CD4 cell counts, how they are clinically and what risk there is….”*

*“we also check the stage (of AIDS) they are in….”*

*“I referred them to their private doctor so he may tell them whether they can have a child or not.”*

It seems as far as these HPs are concerned HIV positive people have to fulfil and adhere to certain conditions determined by the health professionals before they can think of having children. Medical considerations seem to be paramount among these HPs.

### 3.5. The Antichildbearing Stance

The antichildbearing stance is against HIV positive people having children at all. The proponents of this stance regard childbearing by people living with HIV as an unnecessary risk to the unborn child. Their concern is the wellbeing of the child more than anything else. As far as they are concerned it is not only irrational but also immoral for HIV positive people to have children. It is immoral because there is a possibility of having a positive child and thus causing suffering to an “innocent soul” when this can be avoided by not having a child at all. The HPs who take this stance in a way argue that if it is irrational to allow the worst outcome of our actions, and if it is immoral to cause suffering, then it is irrational and immoral for HIV positive people to have children [14]. Four of the HPs interviewed displayed antichildbearing sentiments. Here are some of their responses regarding childbearing among those who are infected:

*“..this is a problem (having children). It will not only be a problem to them but also to the children. The painful thing is to see the children when they get sick of AIDS…that is painful especially if you deal with children who are HIV positive which is what I do most of the time” (counsellor).*

*“I do not think it is necessary (to have a child)—it's not necessary. I believe there can still be a happy marriage without children and perhaps my opinion is heavily influenced by my medical background. I wish I could come out of it and stand on neutral ground, but I do not think it's necessary, they should not” (doctor).*

*“…both of you, you are ill now and the child will be ill as well and the child will be in and out of hospital now and again…or you have a child and five years down the line both of you die what will happen to the child and worse if the child is positive as well, even if its negative what happens to her, and so forth” (nurse).*

*“I really feel pity especially for the children. They are very innocent but they are suffering” (nurse).*

The response of this group to pregnancy among women on treatment or to couples/individuals who intend to reproduce is usually anger, disappointment, dejection, and a feeling of defeat and failure.

### 3.6. The Prorights Stance

What underlies this discourse is a human rights approach to reproductive decision making. Its advocates argued that it is the right of every human being to choose freely without fear or fetters. It is also everyone's right to be given the correct and complete information regarding reproduction when they need it. They argued that

*“every human being has a right, has every right to decide what he wants concerning his health, family, just about everything. It should be his decision. As we have here at OI clinics there are people who choose not to take ARVs, that is the choice of the individual, it is his right…” (male counsellor).*

Another counsellor argued that due to the number of adults infected with HIV in Zimbabwe and given the high chances of having a negative child people have the right to choose to reproduce freely. He said

*“it is very important, to me I think it is very important for HIV positive people to have children because even if I give you our statistics in Zimbabwe, it says around 20% of the adult population are people living with HIV. That is a substantial number if you look at it. So considering the chances of these HIV positive people having negative children if they take the necessary drugs I think there is no reason for them not to have children. So I think being HIV positive should not ever be used to stop someone from having children.”*

## 4. Discussion

Study findings revealed that there are three dominant discourses on the issue of childbearing by HIV positive people among health professionals. Prevalent in the conditional choice and antichildbearing discourses is the view of HIV positive people as “abnormals” who do not have the same reproductive rights and freedom as “normals.” From these perspectives HIV/AIDS is framed as a disability and like most people with disabilities HIV positive people find themselves in a position where their condition is viewed as a handicap. Persons with disabilities often face serious discrimination based on attitudes, perceptions, misunderstandings, and lack of awareness about disability [15, 16]. Similarly people with HIV/AIDS who desire to reproduce usually face discrimination as a result of negative social constructs of HIV/AIDS. As Asch and Fine (1988) note, the attitudes and structural barriers of the nondisabled turn disabilities into handicaps [17]. People with disabilities are usually assumed to be unfit for parenthood. Ferri and Gregg (1998) argue that the reasoning behind such a stereotype is the fear that people with disabilities will produce “defective” offspring [18]. In the case of HIV positive people the fear seems to be not only that they will produce infected children but also that they will increase the number of orphans and child headed households in the society due to their early death. As a result people with HIV/AIDS find their right to reproduce being questioned and sometimes denied by both the medical fraternity and the society. There seems to be a view in this school of thought that the information given to HIV positive people should make them arrive at what is seen as an ideal decision, that is, not to procreate. Such an attitude may discourage HIV positive people from consulting HPs on issues relating to their sexual and reproductive health and life and this may consequently lead to negative health outcomes for HIV positive people.

The prorights stance argues that like any “normals,” HIV positive people have the right to do what they want, when they want, in the matter of reproduction. The fact that they are positive does not make them any less human. This perspective does not frame HIV/AIDS as a disabling factor but rather a chronic but manageable condition. However the consideration of HIV positive people as “abnormals” in the society in general and in the medical fraternity in particular means that HPs with such a liberal view are likely to be few. As a result their influence on the reproductive lives of people with HIV/AIDS is also likely to be limited.

There is also a tendency among most HPs to overemphasise the risk to the child though they are aware that this risk is considerably reduced with the help of prophylactic drugs. The way the risk to the child and the mother is emphasised makes it loom larger in the minds of HIV positive people than it actually is given the fact that HPs are considered as authorities in this field by HIV positive people [11]. There was a tendency among HPs to omit information on strategies to reduce reinfection as well as MTCT when giving HIV positive people information regarding reproduction. The information on how they can lower the rate of reinfection and the incidence of MTCT and thus increase their chances of getting an HIV negative child was usually not given prominence. In their accounts of the reproductive information given to people, ten of the twelve HPs interviewed were silent on the role of nevirapine and other ARVs in lowering the incidence of MTCT and how those who want children can take advantage of this. When probed on their silence on ARVs one counsellor quipped, “…people should not get pregnant because of the availability of ARVs.” True as this may be, HIV positive people deserve to know all the possibilities available to them and it is the duty of HPs to avail that information to them.

Health professionals' attitudes towards childbearing among HIV infected couples were largely shaped by their medical background. The issue of reproduction tended to be medicalised and the aspects of patients' lives and experiences that transcended the biomedical milieu tended to be neglected [11, 19]. Health professionals in the study were more concerned about the potential adverse effects of pregnancy, reinfection, and drug resistance than with the personal and social meaning of childbearing to their patients or their reproductive rights. This tendency to lean towards the biomedical model of health which by its nature is concerned more about the physical rather than the emotional and social health meant that there was a tendency among most health professionals to prescribe rather than advise a course of action.

Many health professionals in the study felt that deciding to have a child required “careful planning and consideration, with a “right time” to fall pregnant, which included an adequately high CD4 count, access to ART and PPTCT programmes, and whether the individual was physically healthy” [11]. Most HPs felt that the “right time” was supposed to be determined by them or by some other health professionals. While the physical health of the infected is important, their psychosocial needs were usually ignored by this dominant discourse. The psychosocial aspect of health is as important as the physical in the overall health of the individual.

The study results indicate that the stance of most HPs in the area of reproduction among people with HIV is that of interested parties who instead of giving value free information give value laden, authoritative advice. The information given to HIV positive people generally emphasises safe sex and the risks involved in pregnancy while discounting the low risk of having a positive child when one is on HAART. Recent studies have indicated that MTCT risk is very low among women on HAART and takes nevirapine at the onset of labour [5, 6]. There seems to be a general tendency among the HPs to commit the error of omission when giving HIV positive people reproductive information. An analysis of conversations with HPs reveals possible reasons for this. These are the principles of medical ethics, the OI clinic's criteria on ARV treatment, the biomedical underpinning of the field of medicine, and to a certain extent the personal views of HPs on reproduction by HIV positive people.

The biomedical model of health is by its nature prescriptive and it views the HP as the authority in terms of HP-patient relationship [20]. Modern medicine, argues Samson (1999), is based on a mechanistic, materialist view of the body and the HP, as the professional, exercises control over this body [9]. The unequal relationship between health professionals and their patients—where the doctor is in a position to determine how a health problem should be dealt with and the patient is not—has come to be accepted as “normal.” People normally do not talk back to the HPs; they just listen passively. As one nurse pointed out during the research,

*“people generally have an impression that a nurse is someone who would just instruct you to do this and that. Now we have a challenge to change the whole process so that people can be able to approach us freely….”*

The interaction between HPs and HIV positive people is embedded in a social and political context in which HPs have medical knowledge and technical expertise that patients usually lack. As a result of their position as “gatekeepers” and because of the power they exercise over the behaviour of their patients they may feel obliged to prescribe rather than to advise their patients [10].

The biomedical perspective taken by most HPs in this research may also be based on a genuine concern for the health and wellbeing of HIV positive people as well as for children born to them and a “misguided” view that curtailing the reproductive rights of HIV positive people is ethical. Until recently, HIV/AIDS has been a fatal disease and still is a potentially lethal and infectious condition [7]. Thus the concern and involvement of HPs in the reproductive decision making process of HIV positive people may emanate from a rational logic to safeguard the health of HIV positive people. Nonetheless, however altruistic their aims may be, the deliberate omission of important information relating to childbearing displayed by some HPs in the study is not medically or ethically justifiable.

## 5. Study Limitations

As noted above this study was conducted in two urban opportunistic infections clinics that were fairly well resourced. As a result of the specificity of this study it possibly may not be representative of the views and attitudes of the majority of health professionals in other urban, nonurban, or resource poor settings.

## 6. Conclusion

The results of this study indicate a general negativity towards reproduction by HIV positive people among health professionals. Most of the reproductive information and advice given to HIV positive people was geared towards discouraging them from childbearing. The information was neither unbiased nor complete and the advice given to HIV positive people was not undirective or neutral as is required of counsellors [13]. The study results also reveal a lack of clear guidelines in dealing with the issue of reproduction among people with HIV and hence the existence of varying discourses on the issue and the haphazard advice given to the patients by different HPs. Though health professionals are entitled to their own ideologies there is a need for clear counselling guidelines for HPs on how to deal with HIV positive people who need to reproduce so as to harmonise the advice given to them. This need is urgent as the number of HIV positive people who intend to reproduce is likely to increase as more people are admitted into the highly active antiretroviral therapy program [21].

## References

[1] Nakayiwa S., Abang B., Packel L. (2006). Desire for children and pregnancy risk behavior among HIV-infected men and women in Uganda. *AIDS and Behavior*.

[2] Kirshenbaum S. B., Hirky A. E., Correale J. (2004). ‘Throwing the dice’: pregnancy decision-making among HIV-positive women in four U.S. cities. *Perspectives on Sexual and Reproductive Health*.

[3] Paiva V., Ventura Filipe E., Santos N., Novaes Lima T., Segurado A. (2003). The right to love: the desire for parenthood among men living with HIV. *Reproductive Health Matters*.

[4] Chen J. L., Phillips K. A., Kanouse D. E., Collins R. L., Miu A. (2001). Fertility desires and intentions of HIV-positive men and women. *Family Planning Perspectives*.

[5] Mykhalovskiy E., Mccoy L., Bresalier M. (2004). Compliance/adherence, HIV, and the critique of medical power. *Social Theory & Health*.

[6] Boer K., Nellen J. F., Patel D. (2007). The AmRo study: pregnancy outcome in HIV-1-infected women under effective highly active antiretroviral therapy and a policy of vaginal delivery. *An International Journal of Obstetrics and Gynaecology*.

[7] Wilcher R., Cates W. Reproductive choices for women with HIV. http://www.who.int/bulletin/volumes/87/11/08-059360/en/.

[8] Hopkins K., Barbosa R. M., Knauth D. R., Potter J. E. (2005). The impact of health care providers on female sterilization among HIV-positive women in Brazil. *Social Science and Medicine*.

[9] Samson C., Samson C. (1999). The physician and the patient. *Health Studies: A Critical and Cross-Cultural Reader*.

[10] Shelton J. D. (2001). The provider perspective: human after all. *International Family Planning Perspectives*.

[11] Harries J., Cooper D., Myer L., Bracken H., Zweigenthal V., Orner P. (2007). Policy maker and health care provider perspectives on reproductive decision-making amongst HIV-infected individuals in South Africa. *BMC Public Health*.

[12] Ko N. Y., Muecke M. A. (2006). Prevailing discourses among AIDS care professionals about childbearing by couples with HIV in Taiwan. *AIDS Care*.

[13] Bor R., Miller R., Goldman E. (1993). *Theory and Practice of HIV Counselling: A Systemic Approach*.

[14] Häyry M. (2004). A rational cure for preproductive stress syndrome. *Journal of Medical Ethics*.

[15] Hanass-Hancock J., Nixon S. A. (2009). The fields of HIV and disability: past, present and future. *Journal of the International AIDS Society*.

[16] Elliott R., Utyasheva L., Zack E. (2009). HIV, disability and discrimination: making the links in international and domestic human rights law. *Journal of the International AIDS Society*.

[17] Asch A., Fine M., Fine M., Asch A. (1988). Introduction: beyond pedestals. *Women with Disabilities: Essays in Psychology, Culture and Politics*.

[18] Ferri B. A., Gregg N. (1998). Women with disabilities: missing voices. *Women’s Studies International Forum*.

[19] Ndlovu V. (2009). Considering childbearing in the age of highly active antiretroviral therapy (HAART): views of HIV-positive couples. *Journal of Social Aspects of HIV/AIDS Research Alliance*.

[20] Minkoff H. (2003). Human immunodeficiency virus infection in pregnancy. *Obstetrics and Gynecology*.

[21] Mutasa-Apollo T. Scaling up treatment in Zimbabwe: the path to high coverage. http://apps.who.int/hiv/events/2013/2_apollo_scaling_up_zim_ias_v_3_2.pdf.

